# Establishing Functional Retina in a Dish: Progress and Promises of Induced Pluripotent Stem Cell-Based Retinal Neuron Differentiation

**DOI:** 10.3390/ijms241713652

**Published:** 2023-09-04

**Authors:** Nonthaphat Kent Wong, Shea Ping Yip, Chien-Ling Huang

**Affiliations:** 1 Department of Health Technology and Informatics, The Hong Kong Polytechnic University, Hong Kong, China; kent.n.wong@connect.polyu.hk; 2 Centre for Eye and Vision Research (CEVR), Hong Kong Science Park, Hong Kong, China

**Keywords:** iPSC differentiation, retinal organoids, retinal ganglion cells, photoreceptors, retinal neurons

## Abstract

The human eye plays a critical role in vision perception, but various retinal degenerative diseases such as retinitis pigmentosa (RP), glaucoma, and age-related macular degeneration (AMD) can lead to vision loss or blindness. Although progress has been made in understanding retinal development and in clinical research, current treatments remain inadequate for curing or reversing these degenerative conditions. Animal models have limited relevance to humans, and obtaining human eye tissue samples is challenging due to ethical and legal considerations. Consequently, researchers have turned to stem cell-based approaches, specifically induced pluripotent stem cells (iPSCs), to generate distinct retinal cell populations and develop cell replacement therapies. iPSCs offer a novel platform for studying the key stages of human retinogenesis and disease-specific mechanisms. Stem cell technology has facilitated the production of diverse retinal cell types, including retinal ganglion cells (RGCs) and photoreceptors, and the development of retinal organoids has emerged as a valuable in vitro tool for investigating retinal neuron differentiation and modeling retinal diseases. This review focuses on the protocols, culture conditions, and techniques employed in differentiating retinal neurons from iPSCs. Furthermore, it emphasizes the significance of molecular and functional validation of the differentiated cells.

## 1. Introduction

The human eye, a vital sensory organ, enables distant sense and vision perception of the world. Extensive research has enhanced our understanding of eye anatomy and function, but it has also uncovered numerous retinal diseases that can impair vision or cause blindness [1]. Retinal diseases damage the retina, which is responsible for converting light into brain-transmitted signals, leading to irreversible retinal cell apoptosis and vision impairment. Common degenerative retinal diseases, including retinitis pigmentosa (RP), diabetic retinopathy, glaucoma, and age-related macular degeneration (AMD), are major causes of global blindness [2,3]. The prevalence of these disorders has risen due to population growth and increased life expectancy [2,3].

Despite progress in retinal development research and clinical investigations, current approaches have not been successful in reversing retinal dystrophy or restoring vision [4,5]. Cellular and animal models, though extensively used, do not fully replicate the human conditions due to morphological, physiological, and molecular differences across species [6,7]. Moreover, limited access to specific eye tissues or cells from deceased patients poses ethical and legal challenges, hindering research on eye diseases. To overcome these obstacles, researchers have turned to stem cell-based approaches, aiming to generate distinct populations of retinal cells for cell replacement therapies and in vitro disease modeling [8]. 

Induced pluripotent stem cells (iPSCs) offer self-renewal and the potential to differentiate into various cell types. The induction of iPSCs from murine embryonic or human adult fibroblasts was first achieved in 2006 through the introduction of four transcription factors [9]. iPSCs exhibit gene expression, epigenetic profiles, and developmental potential similar to embryonic stem cells (ESCs). Since their discovery, iPSCs have proven promising for studying human retinogenesis stages [10,11,12]. iPSC-derived retinal cells exhibit the temporal order of embryonic retinal development and replicate the cellular mosaics and lamination of the human retina, which provides a more realistic model for retinal disorder investigations [12,13,14,15,16,17]. Furthermore, iPSCs derived from patients’ somatic cells could provide the exact genetic and cellular context to study disease-specific mechanisms. This capability has future implications for investigating retinal tissue regeneration and targeted therapies [15,18,19,20].

Recent advances in stem cell technology have facilitated the production of various retinal cell types, including retinal neurons, glial cells, and retinal pigment epithelium (RPE) cells [13,21,22,23,24]. Retinal neurons, particularly retinal ganglion cells (RGCs) and photoreceptors, have received significant attention. RGCs are the first retinal neurons generated and play a critical role in transmitting visual information to the brain, and photoreceptors detect light and initiate visual transduction. Additionally, stem cell-derived retinal organoids, three-dimensional (3D) tissue models, have emerged as valuable tools for studying retinal neuron differentiation and modeling retinal diseases in vitro [25,26,27,28]. By utilizing retinal organoids, RGCs, or photoreceptors derived from stem cells, researchers can investigate the impact of extrinsic and intrinsic factors on retinal neuron differentiation and identify new targets for therapeutic interventions in retinal diseases [25,26,29,30,31]. 

**Figure 1:**
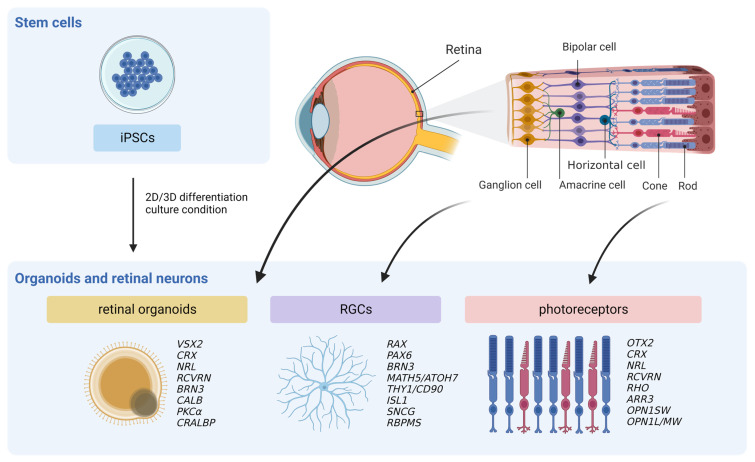
A schematic diagram showing the focus of this review. This review summarizes various culture conditions and techniques of retinal neuron differentiation from iPSCs, with a particular focus on retinal organoids, RGCs, and photoreceptors. Some commonly used molecular marker genes are listed adjacent to the corresponding organoids or cells. iPSCs, induced pluripotent stem cells; RGCs, retinal ganglion cell. Created with BioRender.com (accessed on 27 August 2023).

This review provides a comprehensive overview of the protocols, culture conditions, and techniques employed in differentiating and developing retinal neurons using pluripotent stem cells (PSCs), with a specific focus on retinal organoids, RGCs, and photoreceptors (Figure 1). Additionally, we discuss the molecular and functional validation of the differentiated cells, including prominent molecular markers and assays used to assess the identity and functionality of retinal neurons. Our objective is to summarize the informative aspects of this field and highlight recent key advancements, with the review serving as a valuable resource for researchers and clinicians interested in generating retinal neurons and fostering further progress in this area.

## 2. Retinal Neuron Differentiation from iPSCs

### 2.1. Differentiation of iPSC-Derived Retinal Organoids

Retinal organoids, which closely resemble developing retinal tissue, are 3D self-organizing structures derived from PSCs. They consist of a diverse range of retinal cell types, including RGCs, photoreceptors, horizontal cells, amacrine cells, bipolar cells, and more, making them an invaluable model for studying retinal development and disease modeling in vitro. Though numerous protocols exist for generating retinal organoids, variations in methodology can lead to discrepancies in the composition and maturity of the resulting organoids (Table 1). In this section, we thoroughly review and discuss recent research articles detailing various retinal organoid differentiation protocols, highlighting disparities in culture conditions, differentiation factors, maturation duration, and cellular composition. A summary of experimental features of the selected studies is shown in Table 1.

#### 2.1.1. Generation of Storable Retinal Organoids under Chemically Defined Conditions

To address the need for clinical translation, Reichman et al. developed a straightforward approach for generating storable retinal organoids using completely defined Xeno-free and Feeder-free conditions [16]. This two-step protocol eliminates the need for embryoid body formation. Initially, neuroretinal-like structures containing retinal progenitor cells (RPCs) are generated from iPSCs. Subsequently, early retinal organoids are isolated, and the maturation process occurs during subsequent floating culture. The protocol minimizes the use of exogenous differentiation factors by relying on endogenously produced Dickkopf-related protein 1 (DKK1) and Noggin, induced by iPSCs, which are critical for self-formation of retinal structures [38]. The protocol incorporates N2 supplement, which contains recombinant human insulin, to mimic the role of insulin-like growth factor 1 (IGF-1) in promoting differentiation toward the retinal lineage [39,40]. Moreover, the floating retinal organoids are cultured in ProB27 medium supplemented with fibroblast growth factor 2 (FGF2), favoring neural retinal differentiation [41]. This allows RPCs to spontaneously differentiate into various cell types, faithfully recapitulating the complex spatiotemporal pattern of retinal development. Notably, RGCs emerge around day 35, followed by horizontal and amacrine cells. By day 100, the retinal organoids contain nearly all retinal cell types, including mature photoreceptors, bipolar cells, and Muller glia.

#### 2.1.2. The Role of Retinoic Acid in Retinal Organoid Differentiation

Accumulating evidence from both in vivo and in vitro studies highlights the significance of retinoic acid (RA) in retinal development and photoreceptor determination [42,43,44,45]. However, Li et al. demonstrated that RA was not essential for retinal organoid differentiation, as their differentiation protocol did not include RA supplementation throughout the process [32]. Using iPSCs derived from urine cells, they successfully generated retinal organoids that faithfully recapitulated major steps of retinogenesis including the formation of laminated neural retina, optic vesicles, and mature retinal organoids. Remarkably, their retinal organoids exhibited highly mature photoreceptors encompassing all subtypes, including rods, red/green cones, and blue cones, indicating that exogenous RA supplementation is not required for specifying retinal cell fate or photoreceptor development during retinal organoid differentiation from iPSCs.

In contrast, Sanjurjo-Soriano et al. investigated the effects of RA on initial photoreceptor differentiation and maturation in iPSC-derived retinal organoids [37]. They compared a 2D–3D differentiation protocol with or without the addition of RA at a specific stage. Their findings demonstrated that RA treatment delayed initial photoreceptor differentiation by modulating photoreceptor gene expression. However, it ultimately resulted in highly structured mature retinal organoids containing various retinal cell types, including mature photoreceptors. Notably, the brush border on the surface of retinal organoids, resembling inner segments and outer segment-like structures of the photoreceptors, appeared on day 100 without RA supplementation. In contrast, retinal organoids treated with RA showed the appearance of nascent brush border on day 150. Despite the delay in photoreceptor gene expression, RA supplementation promoted the generation of rod-rich organoids and preserved the outer nuclear layer. The retinal organoids with RA treatment exhibited a well-structured and stratified distribution of the photoreceptor layer with a predominant population of rods (rod–cone ratio 3:1), resembling the parafoveal region of human retina [46]. In contrast, the absence of RA resulted in retinal organoids with a less organized photoreceptor layer and a rod–cone ratio of 1:1. These findings unveiled an additional role of RA in promoting the development of a more organized photoreceptor layer beyond inducing rod photoreceptor differentiation.

Additionally, the study conducted a comparison of differentiation conditions with or without the addition of fetal bovine serum (FBS), which is known to promote lamination of retinal organoids [11]. The results confirmed that the protocol without FBS failed to produce laminated organoids containing a presumptive outer nuclear layer. Furthermore, the differentiation protocol included the addition of taurine on day 42 to further facilitate the lamination and survival of retinal organoids. Overall, the findings underscore the importance of RA and FBS in the differentiation and maturation of iPSC-derived retinal organoids.

#### 2.1.3. Enhancement of Rod Photoreceptor Differentiation in Retinal Organoids through 9-cis Retinal Supplementation

To explore an alternative approach to the widely used all-trans retinoic acid (ATRA), Kaya et al. investigated the impact of 9-cis retinal treatment during retinal organoid differentiation [33]. Their findings revealed that the addition of 9-cis retinal facilitated rod photoreceptor differentiation, promoting a higher proportion of cells adopting a photoreceptor cell fate. By day 120, increased expression of rhodopsin (RHO) and more mature mitochondrial morphology were observed. Notably, this protocol employed a unique step in which medium changes were performed under dim red light and in a dark condition. The precautionary measure aimed to prevent the isomerization of 9-cis retinal to all-trans retinal, allowing 9-cis retinal to be oxidized into 9-cis retinoic acid inside the cells [33]. Unlike ATRA, which exclusively binds to retinoic acid receptors, 9-cis retinoic acid serves as an activator for both retinoid X receptors and retinoic acid receptors, thereby promoting photoreceptor development. As a result, 9-cis retinal supplementation leads to a stronger activation of rod photoreceptor differentiation when compared to ATRA [47,48].

#### 2.1.4. Effects of BMP4 Treatment on the Yield and Cellular Composition of Organoid Differentiation

Capowski et al. examined the influence of bone morphogenetic protein 4 (BMP4) treatment on retinal organoid generation [34]. They differentiated several iPSC lines into retinal organoids and compared cultures with or without a single dose of BMP4 treatment on day 6. Subsequently, they progressively diluted the BMP4 by replacing half of the media on days 9, 12, and 15. Their results revealed that the addition of BMP4 resulted in the appearance of more distinct optic vesicles in the culture plate, leading to a six-fold increase in optic vesicle production when compared to the control group. This finding suggests that an early BMP4 pulse can enhance retinal organoid differentiation, which aligns with a previous report demonstrating increased optic vesicle production in human ESCs (hESCs) with early BMP4 exposure [49].

Berber et al. employed three different protocols for retinal organoid differentiation [36]. The first protocol primarily utilized small molecules for neural and retinal induction. The culture medium included IWR-1e, a Wnt response inhibitor compound, until day 7 to induce retinal differentiation. From day 10 to day 18, the medium was supplemented with smoothened agonist (SAG), a Hedgehog agonist, to enhance neural cell survival. ATRA was introduced to the medium after day 200. Additionally, the medium was supplemented with the Notch signaling inhibitor DAPT from day 29 to day 42 to improve photoreceptor yield. The second protocol involved switching to a different retinal differentiation medium with the addition of HEPES on day 23 and ATRA from day 63 to day 90. The third protocol was similar to the second one, but with the additional inclusion of BMP4 on day 6. Results indicated that organoids generated using the first protocol lacked proper lamination, and the third protocol yielded more mature organoids. Moreover, the study demonstrated that the addition of BMP4 significantly increased the production and quality of retinal organoids per differentiation [36]. It is worth noting that successful retinal organoid formation was still possible without BMP4 supplementation. However, the inclusion of BMP4 treatment resulted in a higher proportion of BRN3A- and CRX-positive cells, representing RGCs and cone photoreceptors, respectively, while not significantly affecting the generation of other retinal cell types [36]. This finding highlights how a single extrinsic stimulation with BMP4 can profoundly alter the cellular composition of resulting retinal organoids.

#### 2.1.5. Different Approaches for Isolating Optic Vesicle-like Structures during Differentiation

The isolation of optic vesicle-like structures has been commonly employed to extract the desired structures at the neural retina stage for further retinal organoid differentiation and maturation. Various methods have been utilized for this purpose. Capowski et al. utilized an ophthalmic surgical knife to dissect the optic vesicle-like domains [34], whereas Li et al. and Kaya et al. mechanically detached the neural retina domains by using Tungsten needles [32,33]. In the study conducted by Sanjurjo-Soriano et al., scalpel excision was employed to identify and excise neural retina-like structures [37]. However, the meticulous dissection of optic vesicle-like structures is a labor-intensive and time-consuming procedure that demands extensive practice and training.

Regent et al. introduced a simple and efficient method to isolate optic vesicle-like domains from adherent cell cultures, which is less complex than dissection and yields improved results for retinal organoid production [35]. Their approach involved scraping the entire adherent culture by using a cell scraper and subsequently growing the cell aggregates in a free-floating condition. This method eliminates the need for subjective selection of optic vesicle-like domains since the entire adherent culture is scraped off. The study reported a larger harvest of retinal organoids with this method, which demonstrated a 2.5- to 4.6-fold increase in retinal organoid production when compared to the dissection method. Furthermore, the proportion of retinal organoids containing one or more pigmented domains was higher with the scraping method [35]. These findings offer an alternative approach to enhancing the efficiency of retinal organoid differentiation.

### 2.2. Retinal Ganglion Cell Differentiation from iPSCs

RGCs are neural cells located in the inner retina and are responsible for transmitting visual information from photoreceptors to the brain through their axons, forming the optic nerve. RGC degeneration is associated with various retinal diseases, including glaucoma and optic neuropathies, which can lead to vision loss. The differentiation of functional human RGCs from PSCs not only provides a valuable resource for studying RGC development, but also holds promise for future cell replacement therapies. This section reviews and discusses several RGC differentiation protocols with a focus on comparing different culture methods and conditions. A summary of experimental features of the selected studies is shown in Table 2.

#### 2.2.1. Generation of Functional RGCs under Chemically Defined Culture Conditions

Teotia et al. developed a chemically defined protocol for generating functional RGCs from iPSCs [50]. The differentiation process involved stepwise recapitulation of developmental mechanisms, starting with neural induction toward RPCs and then progressing through a three-stage differentiation toward the RGC lineage. In the first stage, fibroblast growth factor 8 (FGF8) was added to transiently activate FGF signaling, facilitating initial RGC differentiation in the central retina [59,60]. Activation of the Shh signaling pathway contributed to a similar initiation effect, but beyond this stage, Shh enhanced cell proliferation and maintained RPCs for further differentiation [61]. Coordinated inhibition of the Notch signaling pathway was also critical for commitment along the RGC lineage [62,63], so the γ-secretase inhibitor DAPT was added to downregulate Notch signaling. In the second stage, FGF8 was removed, and cyclopamine was added to further inhibit Shh signaling, which could interfere with RGC differentiation. To promote RGC maturation and reduce cell death in the third stage of differentiation, a combination of brain-derived neurotrophic factor (BDNF), neurotrophin-4 (NT4), and ciliary neurotrophic factor (CNTF) was added to the culture medium. These factors have been shown to inhibit programmed cell death in RGCs and enhance their survival [64,65,66]. The culture medium also included forskolin and Y-27632 as general cell survival promoters to enhance cell viability during maturation [64,67]. This study demonstrated that functional RGCs could be generated from iPSCs using stage-specific chemically defined conditions, allowing precise control and reducing experimental variations [50].

Lee et al. utilized a different approach to initiate retinal differentiation by targeting BMP and transforming growth factor beta (TGF-β) signaling using small molecules called dorsomorphin and SB431542, respectively [51]. Dorsomorphin, a BMP inhibitor, induced eye field progenitors [68], and SB431542, a TGF-β inhibitor, enhanced neural differentiation from PSCs [69]. Additionally, XAV939, a potent tankyrase inhibitor, was added to inhibit canonical Wnt signaling [70], facilitating anteriorization of the neuroectoderm and eye field specification. IGF-1 was employed to promote eye field specification based on a previous report showing its positive regulation of eye and head induction in Xenopus [71]. Results indicated that the addition of IGF-1 enhanced anteriorization and eye field fate. Subsequent co-treatment with IGF-1 and XAV939 demonstrated that the cooperation of IGF activation and Wnt inhibition further facilitated eye field specification. Furthermore, BDNF was supplemented from day 16 until the end of differentiation to improve neuron survival [51]. In summary, their protocol employed a combination of small-molecule inhibitors and growth factors to induce RGC differentiation.

#### 2.2.2. Enhancing RGC Differentiation and Neurite Outgrowth through Biomaterial Incorporation

To improve RGC differentiation efficiency, Chen et al. employed a strategy including the application of a biocompatible scaffold made of polybenzyl glutamate (PBG) [53]. PBG is a peptide-based polymer best known for its biocompatibility and lack of immune response [72,73]. The PBG scaffold was synthesized into a 3D structure by electrospinning, and glutamate was incorporated to stimulate neuron growth [74]. Results demonstrated that the PBG scaffold enhanced iPSC differentiation toward the retinal lineage in a shorter period with the derived RGCs exhibiting improved neurite outgrowth guided by the scaffold [53]. Their protocol involved initiating neural induction using the small molecules IWR-1e, CHIR99021, and SAD, all known to promote neural retinal formation [75]. RGC specification was achieved through the use of RA and BDNF. RA, a primary metabolite of vitamin A, acts as an inducer of neuronal differentiation in the central nervous system and promotes the survival of differentiating RGCs while reducing apoptosis [76,77]. BDNF was added to enhance neural cell development efficiency, accelerate synaptic response development in the early stages of differentiation, and support long-term synaptic activity [78]. Additionally, FBS was included to enhance proliferation efficacy during RGC differentiation with its concentration increased during the adhesion culture period to promote cell attachment [53]. Overall, the incorporation of the PBG scaffold, in conjunction with small molecules and growth factors, offers a promising approach to enhancing RGC differentiation efficiency.

#### 2.2.3. Efficient Protocol for RGC Generation through Dual SMAD and Wnt Inhibition

Chavali et al. presented an efficient two-stage protocol for generating RGCs from iPSCs by employing dual SMAD inhibition and Wnt inhibition [54]. In the first stage, during RPC induction, they utilized LDN193189 to inhibit BMP signaling, XAV939 to inhibit Wnt signaling, and SB431542 to inhibit TGF-β (SMAD) signaling. Previous studies have demonstrated that the inhibition of these pathways enhances the expression of eye field transcription factors (EFTFs) during retinal differentiation [51]. Nicotinamide was also added during RPC differentiation to promote the expression of early eye field markers LHX2 and RAX [54,79]. Furthermore, nicotinamide facilitated cell growth and the development of radial/rosette-like cellular structures [23]. To promote eye field specification of differentiating retinal progenitors, they supplemented differentiation factors IGF-1 and basic fibroblast growth factor (bFGF) [51]. bFGF influences eye field fate by binding to FGF receptors, including FGFR3c, FGFR2c, and FGFR1b, which interact with retinal-inducing FGFs [80].

In the second stage, the RGC differentiation and maturation stage, differentiating cultures were propagated using a cross-hatching technique to dissociate cells into small clusters. Cyclopamine, follistatin, and DAPT were applied to inhibit SHH, TGF-β, and Notch signaling, respectively, to promote RGC maturation. To enhance RGC viability during the expansion phase, cyclic adenosine monophosphate (cAMP), BDNF, NT4, and CNTF were added to the culture medium to promote the growth of neuronal cells [81,82]. NT4 and cAMP function similarly in promoting the structural integrity of axonal branches from differentiated RGCs, resulting in a greater number of axons with more branches [83,84]. CNTF is applied to activate the JAK/STAT3 signaling pathway critical for axon generation through inflammatory stimulation and provide neuroprotection for differentiating RGCs [85]. Additionally, forskolin and Y-27632, a Rho-associated protein kinase inhibitor, were added during the RGC differentiation stage to promote proliferation, lineage commitment, and neurite growth [86]. Overall, this protocol demonstrates the potential for generating functional RGCs from iPSCs with high efficiency through dual SMAD and Wnt inhibition. This protocol has also been employed by other researchers including Gudiseva et al. and Vrathasha et al. [56,57].

#### 2.2.4. Generation of RGCs through Dissociation of Retinal Organoids and Magnetic-Activated Cell Sorting

Rabesandratana et al. employed a unique differentiation approach, first differentiating retinal organoids and then dissociating them before replating the retinal cells onto adherent substrates for further RGC differentiation [55]. The retinal organoid differentiation method was primarily based on the protocol developed by Reichman et al., which utilized retinal differentiation conditions compliant with good manufacturing practices [16]. In their protocol, neural retina differentiation was promoted by applying FGF along with N2 and B27 supplements. After 8 weeks of differentiation, the retinal organoids were dissociated, and the resulting cell suspension was seeded onto culture plates coated with poly-D-lysine and laminin. The dissociated retinal cells were then maintained in retinal differentiation medium for 1 week to enhance RGC maturation. In their study, the authors also explored the possibility of cryopreserving the dissociated cells for potential cell therapy applications. However, their results indicated a significant reduction in cell viability after the freeze–thaw cycle. As an alternative, they thawed the retinal organoids frozen at day 45 of differentiation and further cultured them using the dissociation protocol for RGC production. This approach successfully yielded RGCs without compromising cell viability, offering a viable solution to obtain RGCs quickly from a frozen stock of retinal organoids [55]. In addition, they demonstrated an enrichment of RGCs by employing magnetic-activated cell sorting (MACS) on the dissociated retinal organoid cells, based on the expression of THY1, a widely recognized RGC marker [87]. This sorting approach using MACS and antibodies against THY1 to isolate RGCs was demonstrated in a previous study by Gill et al., which showed an effective enrichment of functional RGCs with electrophysiological responses and mitochondrial axonal transport [88].

#### 2.2.5. A Rapid Protocol for RGC Differentiation Using NGN2 Overexpression

Luo et al. have devised an accelerated protocol for differentiating RGCs from PSCs, which can be completed in approximately one week [58]. Their approach involves the overexpression of neurogenin 2 (NGN2) through lentiviral transduction to expedite the differentiation process. NGN2, a crucial transcription factor within the basic-helix-loop-helix (bHLH) network, plays a pivotal role in regulating cell proliferation, cell identity determination, axonal guidance, and nervous system development [89,90,91]. Importantly, NGN2 is also a regulator of early retinogenesis and RGC specification. Studies on mouse retinal development have revealed that NGN2 expression delineates the forefront of neuronal development, marking the exit of progenitor cells from the cell cycle and their commitment to neural retina fate [92]. In their protocol, lentivirus transduction was performed on day −1, and NGN2 expression was induced on day 0 by adding doxycycline. On day 2, glial cell-derived neurotrophic factor (GDNF) was introduced, followed by a switch to RGC SATO growth medium supplemented with the Notch inhibitor DAPT for the subsequent 4 days. Notch signaling modulation is instrumental in fine-tuning the bHLH network by repressing the expression of ATOH7 and NGN2 [93,94]. Hence, this system enables the directed differentiation of PSCs toward the RGC lineage through both endogenous Notch inhibition and exogenous upregulation of NGN2.

### 2.3. Photoreceptor Cell Differentiation from iPSCs

Photoreceptors, specialized neurons located in the outer retina, play a crucial role in converting light into electrical signals for visual information processing. Dysfunction or loss of photoreceptors can result in vision impairment or blindness, as observed in retinal degenerative diseases like AMD and RP. The generation of photoreceptor cells from human PSCs offers a valuable platform for studying retinal development mechanisms, disease modeling, and potential therapeutic interventions.

Most photoreceptor differentiation protocols share similarities with retinal organoids, encompassing the development of major retinal cell types, but with a focus on the maturation of the photoreceptor cells. However, there are variations in culture conditions among different protocols, and understanding the underlying rationale can help to further enhance or optimize the existing methods. In this section, we provide a summary of recently published protocols for differentiating photoreceptors from human PSCs. A summary of experimental features of the selected studies is shown in Table 3.

#### 2.3.1. Generation of Photoreceptors under Defined Conditions Using Signaling Modulators

In Meyer et al.’s photoreceptor differentiation protocol, the addition of Noggin and DKK1 during iPSC differentiation was employed to inhibit the Wnt and BMP signaling pathways, respectively [19]. These factors have been commonly used in other neural and retinal differentiation protocols in previous research [40,100]. This study demonstrated a positive correlation between the early expression of these factors and the differentiation of anterior neural/eye field fate across different human iPSC (hiPSC) lines, suggesting that the addition of Noggin and DKK1 could enhance retinal differentiation efficiency [19].

Barnea-Cramer et al. aimed to generate photoreceptor cells with higher purity when compared to commonly used embryoid body-based 3D cell differentiation methods [95]. Their protocol involved the direct differentiation of PSCs toward retinal photoreceptor cells by seeding the cells at an extremely low density. By incorporating a single BMP inhibitor, Noggin, in the differentiation medium, they selectively generated PAX6- and CHX10-positive retinal neural precursor cells, which further differentiated into photoreceptor-like cells homogeneously expressing photoreceptor-specific markers, eliminating the need for cell sorting. Additionally, RA, BDNF, CNTF, and DAPT were supplemented in their differentiation protocol. BDNF aided in cell survival and promoted specification toward cone photoreceptor progenitors through the BDNF/TrkB signaling pathway [101,102]. The combination of BDNF and CNTF provided neuroprotection for developing RPCs and maintained photoreceptor survival [103]. DAPT, a γ-secretase inhibitor, was included to inhibit the Notch signaling pathway, driving RPCs out of the cell cycle for differentiation and leading to increased neurogenesis [104]. Moreover, DAPT treatment effectively increased the number of photoreceptors differentiated from stem cells [105].

#### 2.3.2. Photoreceptor Differentiation without Using Extrinsic Signaling Modulators

Zhong et al. developed a protocol that enables iPSCs to differentiate into RPCs and 3D retinal cups without the need for extrinsic signaling modulators such as inhibitors of the Wnt, Nodal, and Notch pathways (e.g., Noggin, DKK1, and DAPT) [12]. Instead, serum, taurine, and RA were added at later stages to enhance cell survival, allowing for prolonged spontaneous differentiation. RA plays a crucial role in the specification and development of retinal photoreceptors [42,106], and previous studies have demonstrated that its impact on photoreceptor differentiation is time- and concentration-dependent [44]. The study also revealed that an extended exposure to relatively high concentrations of RA could potentially hinder photoreceptor maturation. By testing shorter time windows of RA exposure, the researchers observed stronger rod opsin expression, indicating enhanced photoreceptor differentiation [12]. A similar protocol was employed by Gonzalez-Cordero et al. to generate photoreceptors from human PSCs with key mature cell structures such as connecting cilia, nascent outer segments, and presynaptic structures [107]. Importantly, when the resulting cone photoreceptor cells were isolated and transplanted to mouse retina, they were successfully incorporated into the retina and survived, indicating that this differentiation approach produces functional cones and is potentially applicable to therapies for retinal degeneration.

#### 2.3.3. Photoreceptor Differentiation Induced by Small-Molecule Inhibitors

Zhu et al. achieved the generation of retinal cells, including photoreceptors and RPE cells, from iPSCs by using a directed differentiation approach with novel small-molecule inhibitors [96]. In their previous work, they successfully employed Noggin, DKK1, and IGF-1 to inhibit the Wnt and BMP pathways for pluripotent stem cell differentiation into the retinal lineage [13]. In the current protocol, they replaced DKK1 and Noggin with small-molecule inhibitors targeting these signaling pathways, establishing a cost-effective and more chemically defined approach. The ISLI protocol for retinal differentiation comprises the supplementation of the small-molecule inhibitors IWR-1, SB431542, and LDN193189 along with human recombinant protein IGF-1. IGF-1 promotes RPC proliferation by regulating the phosphorylation of AKT and Erk, and it enhances the differentiation potential of RPCs toward retinal neurons including photoreceptors [108]. IWR-1, similar to DKK-1, is an inhibitor of the Wnt signaling pathway, and its application in cell culture has been shown to promote neural differentiation [109]. SB431542 and LND193189 are inhibitors of the TGF-β and BMP signaling pathways, respectively. The ISLI protocol leads to significant downregulation of pluripotency markers and upregulation of eye-field transcription factors within 5 days of neural induction. Importantly, the study demonstrated that the retinal cells derived from iPSCs using the ISLI protocol exhibited characteristics comparable to those generated using the original recombinant protein approach, validating the effectiveness of the ISLI protocol in inducing retinal differentiation while minimizing the variability associated with different manufacturers or batches of recombinant proteins [96].

#### 2.3.4. Enhancing Yield and Maturation of Photoreceptor Cells by Using a Bioreactor

Ovando-Roche et al. employed a stirred-tank bioreactor to enhance the laminar stratification of differentiated retinal cells and optimize the yield of photoreceptor cells [97]. Bioreactors provide improved ventilation and balanced distribution of nutrients through stirring, facilitating the development of complex photoreceptor structures. Previous research has demonstrated that an optimized bioreactor efficiently transfers exogenous factors from the medium to cells without causing tissue damage [110]. In comparison to other protocols, this method exhibited the highest efficiency in photoreceptor differentiation (Table 3). Additionally, this protocol demonstrated enhanced cell proliferation and reduced apoptotic cells. Immunohistochemistry (IHC) results for cell markers were more pronounced, providing stronger evidence of photoreceptor presence [97]. Overall, these findings indicate that the use of a stirred-tank bioreactor holds promise for enhancing photoreceptor cell differentiation. This approach has the potential to maximize photoreceptor cell yield, improve cell survival, and enhance the development of photoreceptor structures.

## 3. Detection of Molecular Markers to Confirm Retinal Cell Identity

In the majority of reviewed articles, immunostaining or immunofluorescence techniques were employed to identify different retinal cell types and assess the expression of specific cell markers to evaluate differentiation efficiency. This section provides an overview of the markers utilized by researchers in retinal differentiation studies. Reference to Table 1, Table 2 and Table 3 will provide further information on these marker genes.

### 3.1. Molecular Markers for Evaluating Retinal Organoid Differentiation

Markers such as PAX6, RAX, LHX2, VSX2 (CHX10), SOX1, and SIX3 have been used to confirm the specification of the eye field during retinal organoid differentiation [16,32]. Positive expression of these markers indicates the differentiation of iPSCs toward the retinal lineage. Additional markers are employed to confirm the cell identity within the retinal organoids. For example, RGCs, the first subtype of cells to develop during retinal organoid differentiation, express markers such as BRN3A, SNCG, Calretinin, and PAX6 [16,34]. AP2, PAX6, calbindin (CALB), and calretinin are used to confirm the presence of amacrine cells, as stated in several articles [16,33,34]. Horizontal cells, the subsequent cell type to develop, can be identified using markers such as LIM1, PAX6, CALB, PROX1, and OC1. PROX1 and OC1 are transcription factors expressed in horizontal cells [16,32,33,34]. Mature photoreceptors are identified using specific cell markers such as S opsin (OPN1SW), L/M opsin (OPN1L/MW), RHO, CRX, and NR2E3 [16,37]. RHO is used to identify rod photoreceptors, OPN1L/MW is used for red/green cone photoreceptors, and OPN1SW is used for blue cone photoreceptors [32]. There are some markers for immature photoreceptors or photoreceptor precursors. CD73, when co-expressed with recoverin (RCVRN), OPN1SW, and OPN1L/MW, serves as a specific marker for photoreceptor precursors. Cone arrestin (ARR3) and RCVRN increase as RPCs commit to the photoreceptor lineage. OTX2 is a marker for immature photoreceptors [16]. Bipolar cells can be identified using markers such as PKCα and CHX10, and G0α is a cell marker for rod and cone ON bipolar cells [16,34]. Muller cells can be identified using the markers SOX9, glutamine synthase, and CRALBP [16,32].

In addition to the aforementioned cell type-specific markers, other markers have been employed in immunostaining to identify particular cell types. Acetylated tubulin, a marker for connecting cilia, has been used to demonstrate the existence of thin structures adjacent to RCVRN+ cells, indicating the formation of cilia and photoreceptor outer segments [16]. Bestrophin and Ezrin were employed as markers to confirm the correct polarization of human iPSC-derived RPE cells in culture. MITF, a key transcription factor specific to retinal pigmented epithelial cells, is used to identify RPE [16]. ZO-1, a tight junction marker observed in epithelial cells, can be found at the apical side of the retinal pigmented epithelium [16,32]. In addition to ZO-1, fibronectin, MCM2, PDE6α, and Gtα-1 were used as markers for different target cells [32]. Fibronectin is present at the basal side of the retinal pigmented epithelium and is used to assess the polarity of neural retina. MCM2, a proliferative marker co-expressed with CHX10 in neural retinal epithelium, and PDE6α and Gtα-1, functional proteins expressed by photoreceptors involved in the phototransduction pathway, were also employed as markers.

ChAT, a marker of early retinogenesis, specifically identifies starburst amacrine cells in the retina, which are an early-born subtype of amacrine cells [34]. Bassoon is used to visualize ribbon synapse in photoreceptors, and CTBP2 and VGLUT1 serve as photoreceptor pre-synaptic markers [33]. ARL13B is used to visualize the outer-segment cilia of photoreceptors, and PCNT is a basal marker for photoreceptors, identifying the basal bodies of photoreceptors [34,35]. Ezrin, Mertk, PMEL17, and AQP1 were also used as immunostaining markers for RPE in the study conducted by Regent et al. [35]. Mertk confirms the identity for RPE as a tyrosine kinase receptor involved in phagocytosis of shed outer segments of RPE. Ezrin and PMEL17 are markers for the pigmented domain of the pigmented epithelium, confirming the identity of RPE. AQP1, when co-expressed with CHX10, serves as a marker for ciliary epithelium and represents the ciliary margin stem cells.

Although retinal organoids contain both RGCs and photoreceptors, more precise markers for their identification will be described in the following section.

### 3.2. Molecular Markers for Evaluating Retinal Ganglion Cell Differentiation

SOX2, a transcription factor involved in neuronal development, is expressed in differentiated RGCs [111]. It is highly expressed in progenitor cells, but down-regulated during the post-mitotic stage [112]. SOX2 is associated with the maintenance of neural progenitor identity, and its absence may result in maturation defects specific to RGCs [113]. RAX and PAX6 are early eye field progenitor markers that are highly expressed in RPCs, and the expression of Rx, encoded by the RAX gene, peaks when the Wnt and BMP signaling pathways are inhibited [54]. RAX promotes the expansion of RPCs and the commitment to retinal cell differentiation, and PAX6 plays a critical role in determining retinal cell fate and axon guidance between retinal cells [114].

CHX10 is a marker for RPCs and should be absent after RPC differentiation [53]. It plays a critical role in progenitor cell proliferation and specification in the developing retina, including the regulation of mosaicism and genetic manipulation [115]. CHX10 guides RPCs to either continue or exit the cell cycle at the G1 phase, thus controlling cell proliferation [116]. LHX2 is an eye field transcription factor used to verify the optic cups [51]. It modulates the SHH signaling pathway during early retinal neurogenesis to promote RGC differentiation [117]. Ki67, another marker for RPCs, is highly expressed in the early stage of organoid development [57]. Its expression decreases as RPCs mature into RGCs, and it is positively correlated with the number of highly proliferative cells [118,119].

THY1, also known as CD90, is a glycophosphatidylinositol-linked glycoprotein expressed on the cell surface of neurons, thymocytes, subsets of fibroblasts, and endothelial cells [120]. THY1 antigen is present in RGCs and the inner limiting membrane of the retina but not in other retinal neurons [87]. This unique expression pattern of THY1 in RGCs makes it a useful marker for selecting RGCs using various cell-sorting techniques [54], and its expression correlates positively with the number of viable RGCs after differentiation [121]. However, as THY1 is not exclusively expressed by RGCs and can also be found in other cell types as mentioned above, it is recommended to incorporate additional markers to achieve a more specific and reliable identification of RGCs.

MATH5, also known as ATOH7, is crucial in determining RGC cell fate and is an essential transcription factor that activates RGC specification, differentiation, and optic nerve generation [50,122]. Studies have shown that the majority of RGCs fail to develop in the absence of Math5 [123]. MATH5 confers competence to RPCs for RGC lineage commitment [124] and regulates the expressions of POU-domain transcription factors including BRN3 family members (BRN3A, BRN3B, and BRN3C) [125]. These transcription factors are expressed in post-mitotic RGCs and play distinct roles in regulating RGC differentiation, dendritic stratification, and axonal projection during RGC development [126,127,128].

BRN3A is specifically expressed by RGCs and is commonly used to identify RGCs in in vitro cultures of mice retinas. It modulates RGC dendritic stratification, and its deletion leads to an alteration in dendritic stratification and the ratio of monostratified and bistratified RGCs [129]. BRN3B is expressed in post-mitotic ganglion cell precursors and is involved in RGC differentiation and axon formation [130]. Approximately 80% of RGC precursors express BRN3B after terminal mitosis, followed by the subsequent expression of BRN3A and BRN3C [129]. BRN3B plays an essential role in the development of RGCs, and its absence results in over 70% RGC loss, as it regulates axon outgrowth, pathfinding, and proper polarization of RGCs [131]. γ-synuclein (SNCG) is highly expressed in the axons and cytoplasm of RGCs, and co-expression of BRN3A and SNCG is a characteristic feature of mature RGCs [132].

Islet1, also known as ISL1, is a LIM homeodomain transcription factor expressed in developing and mature ganglion cells, cholinergic amacrine cells, ON bipolar cells, and horizontal cells in the retina of most vertebrates [133]. It plays an important role in the differentiation, cell specification, and maintenance of phenotypes of retinal cells [134]. ISL1 is believed to regulate the expression of a subset of RGC genes downstream of MATH5, and its expression overlaps with that of BRN3B for a certain period [135]. It represents a distinct branch of the RGC gene regulatory networks downstream of MATH5, intersecting with BRN3B. However, there are functional differences between ISL1 and BRN3B, as many genes expressed in RGCs are regulated by either ISL1 or BRN3B alone, whereas some genes are regulated by other factors [135]. ISL1 also plays a positive regulatory role in sustaining the expression of BRN3B after the decrease in MATH5 expression. Overall, ISL1 is essential for RGC development, and it reveals two distinct intersecting branches of the RGC gene regulatory networks downstream of MATH5, directed by BRN3B or ISL1 [135].

TUJ1 is a cytoskeletal marker for RGCs and a major component of microtubules in neuronal somas, dendrites, and axons [136]. Its expression is measured to quantify RGC somas and axonal bundles [137]. MAP2 is a microtubule-associated protein that helps maintain the microtubule structure in order to preserve neuronal morphology. It could be utilized as another cytoskeletal marker that demonstrates axons and dendrites of RGCs [138]. TAU, an axonal marker, is expressed in matured RGCs, and mutation in TAU can promote RGC apoptosis by enhancing caspase-2 expression [139]. Developing RGCs co-express MAP2 and TAU until they reach definitive maturation. NFM is also an axonal marker that demonstrates the structural components of neurons and is highly expressed throughout differentiation. NFM is measured to confirm the presence of neurites extended from precursor cells in the early stage of differentiation [53].

Tubb3 (βIII-tubulin) belongs to the tubulin protein family that forms microtubules in the cytoskeleton. It is expressed primarily in neurons and is involved in axon guidance and maintenance. Its neural-specific nature has been used to identify neurons within brain tissues, distinguishing them from glial cells that typically lack Tubb3 expression [140,141]. RGCs exhibit high expression of Tubb3 due to their neuronal origin [142], making it suitable for the identification and quantification of RGCs in different optic nerve injury models [143,144].

HuC/D is a marker for immature and mature RGCs that indicates RGC differentiation, and its expression is measured to detect the presence of RGCs [145,146]. RNA-binding protein with multiple splicing (RBPMS) has been used to semi-quantitatively measure the amount of RGCs, since its expression closely relates to RGC immunoreactivity, and its somal diameter is equivalent to the area of RGCs [147,148]. Furthermore, GAP43 is a mature RGC marker essential for guiding axons from the optic chiasm into the optic tracts [149]. CRALBP is a retinoid-binding protein involved in the RPE visual cycle [150]. It is encoded by the RLBP1 gene, and mutations in RLBP1 may lead to autosomal recessive RP [151]. Additionally, CRABLP may play a critical role in chromophore transportation from RPCs to intrinsically photosensitive RGCs (ipRGCs), which are a subset of RGCs with the unique ability to directly sense light themselves. They are involved in visual image formation [152]. OPN4 is a typical gene marker for ipRGCs, and encodes for the protein melanopsin, which is a photopigment primarily expressed in ipRGCs and is essential for light-sensing functions [153]. TBR2 is a T-box-containing transcription factor that has an enriched expression in ipRGCs. It is crucial for regulating the amount of OPN4-expressing ipRGCs [154]. In addition, research has shown that the expression of BRN3B transcription factor could help to molecularly determine the subpopulation of ipRGCs [155].

KCNG4, CB2, and SPP1 are featured markers for α-RGCs [52]. KCNG4 is a potassium channel modulator expressed in the early stage of RGC differentiation, and the expression of KCNG4 and SPP1 marks all four types of α-RGCs [156,157]. CB2 labels OFF-transient α-RGCs and is co-localized with BRN3-positive RGCs [52]. CART, CDH6, and FSTL4 are specific molecular markers for directionally selective RGCs (DS-RGCs). CART and CDH6 classify bistratified ON-OFF DS-RGCs, which respond to the onset and termination of light, and FSTL4 classifies ON DS-RGCs, which only respond to light onset [158].

### 3.3. Molecular Markers for Evaluating Photoreceptor Cell Differentiation

OTX2 and recoverin (RCVRN) are used to demonstrate functional photoreceptors after differentiation. OTX2 is a transcription factor that promotes RHO generation and inhibits the Wnt signaling pathway, which is critical for the renewal of RPCs and the maintenance of stem cell status [159,160]. Photoreceptor cells expressing OTX2 concentrate in the developing outer nuclear layer and subsequently mature. RCVRN serves as a pan-photoreceptor marker and is a calcium-binding photo-transduction protein that is highly expressed in fetal retina [95,161]. It plays a critical role in maintaining the calcium concentration gradient across the retina layer.

CRX is a neuroretinal marker for early-expressed post-mitotic photoreceptors, and NRL is a post-mitotic rod precursor marker that is expressed at later stages [19]. The Rax gene regulates CRX and NRL levels, enhancing the expression of photoreceptor genes (RHO, OPN1SW, and OPN1L/MW), leading to photoreceptor differentiation [162]. CRX is crucial for photoreceptor differentiation, and NRL is required for rod specification [163]. Another study showed that recoverin and opsin are expressed only in CRX+ cells [13]. Loss of NRL results in the absence of rod-specific gene expression and the upregulation of cone-specific genes, leading to cone cell development [164].

The expression of RHO confirms the presence of functional rod cells, which amplify light signals to excite rod cells for twilight vision [165]. S-opsin, M-opsin, and L-opsin indicate the ability of cone cells to transmit light signals for colored vision [166]. They are responsible for perceiving different wavelengths of visible light, specifically blue, green, and red, respectively.

Cyclic nucleotide-gated (CNG) channels guide phototransduction pathways of rod and cone photoreceptors [167]. These channels translate light stimuli into electrical signals, spreading the signal through photoreceptor cells [168]. Mutations in CNGA1 and CNGB1 genes can cause autosomal recessive RP [167]. Additionally, RETGC, ODE6B, and ARR3 are mediators that are critical for light adaptation and photoreceptor desensitization related to G-protein signaling cascades [169,170]. ARR3 is a cone-specific marker, and its expression can be altered by RA to regulate cone cell differentiation [37]. Measuring phototransduction markers indicates the ability of cultured photoreceptor cells to convert light signals into nerve impulses and form images [171].

Furthermore, NR2E3 is a transcription factor that is critical for photoreceptor fate specification and differentiation [95]. In addition to CRX and NRL, NR2E3 is expressed in late retinal progenitors and early precursors. NR2E3 inhibits S-cone expression in human retina, and NRL, a rod-specific transcription factor, regulates RHO expression. Co-expression of NR2E3 and NRL promotes rod development [172]. PDE6α and PDE6β are markers used to measure rod photoreceptors, forming the phosphodiesterase (PDE) protein complex crucial in the phototransduction cascade [173]. PDE6α is concentrated in the rod’s outer segment membrane, and it is activated by transducin during phototransduction [174]. Dysregulation of PDE6α leads to cGMP accumulation in photoreceptor cells, which can be cytotoxic to the retina [174]. Under normal conditions, PDE6α promotes the hydrolysis of cGMP in response to light, resulting in membrane hyperpolarization [175]. On the other hand, PDE6β expression is directly proportional to the rod population, demonstrating the differentiation efficiency of the protocol used [37].

Other markers, such as B lymphocyte-induced maturation protein-1 (BLIMP1), Aryl hydrocarbon receptor-interacting protein-like 1 (AIPL1), and Retinoid X receptor-gamma (RXRγ), have been measured to demonstrate differentiated photoreceptors [96]. BLIMP1 is a photoreceptor marker that regulates structural and secreted proteins in the retina [176]. BLIMP1 stabilizes the cell specification to photoreceptors rather than bipolar cells by downregulating bipolar cell-specific genes [163]. AIPL1 is also a marker for mature photoreceptors, functioning as a chaperone for rod PDE and maintaining cone cell survival [177,178]. Loss of AIPL1 can lead to retinal dystrophies and maculopathy [179]. RXRγ is a cone-specific marker expressed in developing and differentiated cone photoreceptor cells. It is one of the earliest known markers of photoreceptors and is responsible for regulating neuritogenesis [180,181]. RXRγ suppresses S-cone expression by forming a heterodimer with TRβ2, and the dissociation of RXRγ promotes TRβ2 dimerization, leading to M-opsin expression [182].

CD73 and CD133 are cell surface markers expressed in fetal and adult retina. They have been used to distinguish developing rod cells (co-expression of CD73 and CD133) from matured photoreceptors (expressing CD73 only) [97,183,184]. Furthermore, G-protein subunit alpha transducin 1 (GNAT1) is crucial for rod and cone phototransduction. Without GNAT1, rod and cone photoreceptors are incapable of visual perception under light-adapted conditions [185]. In the absence of rod and cone photoreception, the melanopsin-mediated signaling pathway through ipRGCs becomes the sole phototransduction pathway. This pathway regulates the expression of melanopsin, promoting cortical activity in pattern-forming centers in the brain [186]. Therefore, the expression of GNAT1 demonstrates the phototransduction ability of rod and cone cells.

### 3.4. Other Techniques and Functional Assays for Evaluating Retinal Neuron Differentiation

In addition to immunofluorescence staining, researchers commonly use real-time quantitative PCR (qPCR) and flow cytometry analyses to provide supportive information on gene expression during retinal differentiation [37,56,57]. qPCR is a fast and convenient molecular assay that measures gene expression at the transcriptional level. Sanjurjo-Soriano et al. performed qPCR to analyze the expression of transcription factors involved in retinal specification (OTX2, SIX3, RAX, and VSX2) as well as photoreceptor markers (CRX, NR2E3, NRL, RCVRN, GRK1, RHO, ARR3, and OPN1MW) to compare the effects of differentiation protocols with or without RA [37]. On the other hand, flow cytometry can identify and quantify specific cell types based on the expression of cell surface markers. Gudiseva et al. and Vrathasha et al. utilized flow cytometry to characterize their iPSC-derived RGCs based on the highly positive expression of BRN3, SNCG, CD90 (THY1), and RBPMS [56,57].

Single-cell RNA sequencing (scRNA-seq) has emerged as a powerful technique for the comprehensive profiling of individual cells in culture with high resolution. Sridhar et al. applied scRNA-seq to compare single-cell transcriptomes of human fetal retina, hPSC-derived retinal organoids, and retinospheres [187]. Their findings revealed that retinal organoids and retinospheres closely resembled the fetal retina in terms of cellular composition and gene expression patterns. However, some differences were observed in the developmental trajectories of specific cell types, such as photoreceptors, between the organoids and the fetal retina. The authors’ dataset provides a reference to assess the development stages of retinal organoids and their similarity to the fetal retina [187]. Luo et al. compared their iPSC-derived RGCs to the fetal retina and retinal organoids using scRNA-seq [58]. They visualized the similarity of their RGCs to the RGC populations from the fetal retina or retinal organoids using UMAP reduction [188]. Their analyses demonstrated shared marker genes between their RGCs and the datasets, with greater similarity to fetal RGCs than organoid RGCs. For example, their RGCs and fetal RGCs predominantly expressed BRN3A, whereas most organoid RGCs express BRN3B. NEFL and NEFM, axon skeleton markers, were highly expressed in their RGCs and fetal RGCs, consistent with another human fetal retina scRNA-seq study [189]. This highlights the utility of scRNA-seq data for assessing differentiated retinal cells based on gene expression at the global population and specific cluster levels.

Though molecular features provide preliminary characterization of cell type identity, further confirmation of cellular structures and functional assays can provide more solid evidence. Transmission electron microscopy (TEM) is commonly used to study the ultrastructure of retinal cell types, especially photoreceptors, which have organized outer segments packed with photopigments responsible for capturing light and initiating visual signals [190]. TEM has been applied to reveal specific ultrastructures of iPSC-derived photoreceptors, including the outer limiting membrane, outer segment, inner segment rich in mitochondria, connecting cilium, and basal body [32]. TEM analysis has also been employed to compare the maturation of photoreceptors differentiated with 9-cis retinal treatment versus ATRA treatment, showing a more advanced stage of maturation in photoreceptors treated with 9-cis retinal [33]. These applications demonstrate the value of TEM in providing detailed information about the ultrastructure and organization of retinal cell types, validating successful differentiation.

Electrophysiological responses, such as patch-clamp recording and calcium imaging, can demonstrate retinal cell identity and functional capacity beyond morphological and molecular features by testing neuronal function [19]. Patch-clamp recording measures currents through ion channels and receptors in RGCs, shaping action potentials initiated by voltage-gated sodium channels [191]. Membrane depolarization voltage in RGCs reflects retinal signal transduction and should resemble the action potential of native RGCs [192]. Lee et al. demonstrated fast inward sodium currents and outward rectifying potassium currents through voltage-clamp recording in their differentiated neurons, and current-clamp mode revealed spontaneous firings, indicating electrophysiological activity of the RGCs [51]. Multielectrode arrays have also been used to record spiking activity in physiologically identified retinal cells, with large spikes predominantly emitted by RGCs [55].

Calcium imaging is a useful technique for investigating RGC activity. It visualizes ganglion cell firing patterns in response to visual stimuli, as calcium influx often accompanies neuronal activation and spike signaling [193]. Luo et al. utilized calcium imaging to stage RGC maturation and observed calcium entry in immature RGCs upon depolarization induced by a GABA agonist, resembling KCl-induced depolarization [58]. Mature RGCs did not demonstrate a depolarization-induced calcium influx upon the same stimulation. Previous research has demonstrated an excitatory characteristic in immature RGCs upon GABA receptor activation [194]. Calcium imaging provides insights into whether RGCs are fully matured or not.

## 4. Perspectives and Conclusions

The utilization of human iPSCs holds great promise for translating retinal research into clinical applications, as they possess the capacity to differentiate into various retinal cell types. In this review, we have extensively examined different protocols for differentiating retinal neurons from iPSCs. These protocols have made significant advancements in generating specific retinal neurons, particularly RGCs and photoreceptors, which serve as a foundation for developing innovative therapies to restore vision in cases of degeneration or injury.

Remarkable progress has been made in the development of iPSC-based retinal cell replacement therapies, which offer hope for vision restoration through cell transplantation or organoid grafting. Animal studies focusing on retinal degeneration have demonstrated the survival, integration into the host retina, and successful improvement of visual function following transplantation of iPSC-derived photoreceptors or RGCs [57,58,98,99]. These findings suggest that iPSC-derived retinal cells may hold potential for personalized cell therapy in the future. Clinical trials are currently underway to assess the feasibility and safety of implanting specific iPSC-derived retinal cell types in humans [195]. Preliminary results have shown slowed vision loss without adverse effects, although further refinement and optimization of treatment approaches are necessary. Additionally, iPSC-derived retinal neurons can be employed for high-throughput drug screening, facilitating the identification of new molecular targets and the testing of novel therapies [196]. The development of patient-derived retinal organoids has greatly improved the accessibility to retinal tissue for studying the mechanisms of retinal diseases with strong genetic bases. However, regarding multifactorial traits such as AMD or diabetic retinopathy, more complicated culture conditions like co-culturing with RPE or the incorporation of the vascular network might be required to recapitulate physiological interactions [25]. Hence, it is important to acknowledge that organoid technology still has a multitude of limitations that must be considered.

In conclusion, iPSC-derived retinal neuron cells represent a revolutionary approach in translating basic research into drug discovery and treatment strategies for vision loss caused by retinal diseases. Despite the challenges that lie ahead, such as achieving a high purity of cell types, recapitulating the complex retinal architecture, and ensuring the safety of transplantation, iPSC-derived retinal cells provide a solid foundation for ongoing advancements in translating knowledge into clinical applications for the treatment of retinal diseases.

## Figures and Tables

**Table 1:** Experimental features of retinal organoid differentiation.

Authors	Year	Cell Source	Differentiation Procedures	Differentiation Culture Media	Differentiation Factors	Immunostaining Maker	Time Length	Other Assays
Reichman et al. [16]	2017	hiPSC	NR, RO	Essential 6 medium, ProB27 medium	N2 supplement, FGF2	RPC: VSX2, Ki67 Photoreceptor: CRX, NRL, NEUROD1, RCVRN, CAR, RHO, OPN1SW, OPN1L/MW, CD73, OTX2 RGC: BRN3A, PAX6 AC: AP2, PAX6 HC: LIM1, PAX6 BC: PKCα, VSX2 MC: GLUTAMIN SYNTHASE, SOX9	28–280 days	qPCR: Eye field specification: SIX3, MITF, VSX2, PAX6, RAX, LHX2 Photoreceptor lineage: NRL, CRX, NEUROD1, RCVRN, CAR Mature photoreceptor: RHO, OPN1SW, OPN1L/MW RGC: BRN3A, BRN3B AC: GAD2 HC: LIM1 BC: PKCα MC: GLAST1, RLBP1
Li et al. [32]	2018	Urine cell-derived hiPSC	EB, RO	mTeSR1 medium, NIM, RDM	Blebbistatin, FBS, Taurine, GlutaMAX	Eye field fate: SOX1, PAX6, OTX2, LHX2, SIX3 RPC: VSX2(CHX10), MCM2 RGC: BRN3 AC: AP2 HC: PROX1 BC: PKCα MC: CRALBP Photoreceptor: RHO, OPN1SW, OPN1L/MW, RCVRN, PDE6α, GNAT1	6 weeks	RT-PCR: Eye field transcription factor: PAX6, LHX2, RAX, SIX3, SIX6
Kaya et al. [33]	2019	hiPSC	EB, OV, RO	E8 medium, NIM, PIM	Y-27632, FBS, taurine, GlutaMAX, retinoid, N2 supplement, IGF-1, beta-mercaptoethanol, 9-cis retinal, ATRA	RPC: VSX2(CHX10) RGC: BRN3A Pan-photoreceptor: RCVRN Photoreceptor: CRX Rod: RHO Cone: OPN1L/MW, OPN1SW AC/HC: CALB BC: PKCα MC: CRALBP Ribbon synapses: Bassoon	200 days	Western blot: Rod photoreceptor: RHO
Capowski et al. [34]	2019	hiPSC	EB, OV, RO	NIM, RDM	BMP4, FBS, taurine, chemically defined lipid supplement, ATRA	Early retinogenesis: VSX2, Ki67 RGC: BRN3B, SNCG Photoreceptor precursors: OTX2 Photoreceptor: CRX, RCVRN Cone: ARR3, OPN1L/MW, OPN1SW Rod: NRL, NR2E3, RHO Photoreceptor pre-synaptic marker: VGLUT1, CTBP2 HC: OC1 AC: CaR BC: PKCα, GNAO1	175 days	Optical coherence tomography (OCT)
Regent et al. [35]	2020	hiPSC	EB, OV, RO	E8 or mTeSR1 medium, NIM, RIM	Y-27632, B27 supplement without vitamin A, GlutaMAX, IGF-1, FBS, taurine, 9-cis retinal, N2 supplement	RPC: CHX10 RGC: BRN3A Photoreceptor: ARL13B, PCNT, RCVRN Rod: RHO Cone: OPN1SW, OPN1L/MW AC/HC: CALB MC: CRALBP BC: PKCα	200 days	NA
Berber et al. [36]	2021	hiPSC	EB, RO	Method (1) mTeSR Plus medium, BE6.2 medium, LTR medium Method (2&3) mTeSR Plus medium, NIM, RDM, RC2 medium, RC1 medium	Method (1) Blebbistatin, IWR-1e, SAG, ATRA, DAPT Method (2) Blebbistatin, HEPES, ATRA Method (3) Blebbistatin, HEPES, ATRA, BMP4	RPC: VSX2 RGC: BRN3A, SNCG Photoreceptor: CRX, RCVRN Rod: RHO Cone: OPN1SW AC: AP2	85 days	NA
Sanjurjo-Soriano et al. [37]	2022	hiPSC	NR, RO	E6 medium, RDM	Method (1) N-2 supplement, FGF2 Method (2) N-2 supplement, FGF2, FBS, B27 supplement without vitamin A Method (3) N-2 supplement, FGF2, FBS, B27 supplement without vitamin A, taurine, RA	Photoreceptor: CRX, RCVRN, NR2E3 Rod: RHO Cone: ARR3, OPN1SW	85 days	qPCR: ARR3, BRN3A, CRX, GAD2, GLAST1, LIM1, NRL, NR2E3, OPN1MW, OTX2, PKCα, RAX, RCVRN, RHO, GRK1, SIX3, VSX2 Western blot: anti-PDE6B, anti-β-tubulin

AC: amacrine cell; AP2: activating protein 2; ARL13B: ADP-ribosylation factor-like protein 13B; ARR3: arrestin 3; ATRA: all-trans retinoic acid; BC: bipolar cell BMP4: bone morphogenetic protein 4; BRN3A: brain-specific homeobox/POU domain protein 3A; BRN3B: brain-specific homeobox/POU domain protein 3B; CALB: calbindin; CAR: cone arrestin; CRALBP: cellular retinaldehyde-binding protein; CRX: cone-rod homeobox; CTBP2: C-terminal-binding protein 2; EB: embryoid bodies; FBS: fetal bovine serum; FGF2: fibroblast growth factor 2; GAD2: glutamic acid decarboxylase 2; GLAST: glutamate aspartate transporter; GNAO1: G protein subunit alpha O1; GNAT1: G protein subunit alpha transducin 1; GRK1: G protein-coupled receptor kinase 1; HC: horizontal cell; hiPSC: human induced pluripotent stem cell; IGF-1: insulin-like growth factor 1; LHX2: LIM homeobox protein 2; LIM1: LIM homeobox protein 1; MC: Muller cell; MCM2: minichromosome maintenance complex component 2; MITF: microphthalmia-associated transcription factor; NA: not available; NEUROD1: neurogenic differentiation factor 1; NIM: neural induction medium; NR: neural retina; NR2E3: nuclear receptor subfamily 2 group E member 3; NRL: neural retina leucine zipper; OC1: one cut domain, family member 1; OPN1L/MW: opsin 1 cone photoreceptors, medium and longwave-sensitive; OPN1SW: opsin 1 cone photoreceptors, short-wave-sensitive; OTX2: orthodenticle homeobox 2; OV: optic vesicles; PAX6: paired box protein Pax-6; PCNT: pericentrin; PDE6α: phosphodiesterase 6 alpha; PIM: proneural induction medium; PKCα: protein kinase C alpha; PROX1: prospero homeobox protein 1; qPCR: quantitative polymerase chain reaction; RAX: retina and anterior neural fold homeobox; RCVRN: recoverin; RDM: retinal differential medium; RGC: retinal ganglion cell; RHO: rhodopsin; RIM: retinal induction medium; RLBP1: retinaldehyde-binding protein 1; RO: retinal organoid; RPC: retinal progenitor cell; RT-PCR: reverse transcription-polymerase chain reaction; SAG: smoothened agonist; SIX3: sine oculis homeobox homolog 3; SIX6: sine oculis homeobox homolog 6; SNCG: gamma-synuclein; SOX1: SRY-box transcription factor 1; SOX9: SRY-box transcription factor 9; VGLUT1: vesicular glutamate transporter 1; VSX2: visual system homeobox 2.

**Table 2:** Experimental features of RGC differentiation.

Authors	Year	Cell Source	Differentiation Procedures	Differentiation Culture Media	Differentiation Factors	Markers	Time Length	Functional Assays	Efficiency	Tests for Efficiency
Teotia et al. [50]	2017	mESC, hiPSC	EB, neural-rosettes, RPC, RGC	NIM, neural expansion medium	Noggin, DKK1, glutamine, N2, B27, bFGF, IGF-1, SHH, FGF8, DAPT, follistatin, cyclopamine, BDNF, forskolin, NT4, CNTF, cAMP, Y-27632	ATOH7, BRN3B, bIII-tubulin, THY1, GAP43, ISL1, RAX, PAX6, VSX2(CHX10)	58 days	Electro-physiological responses (patch-clamp recording)	84.5 ± 13.0% (ATOH7+) 68.4 ± 18.6% (BRN3+)	ICC
Lee et al. [51]	2018	hiPSC	EB, neural rosettes, RGC	Human ESC medium (-bFGF), N2 medium, N2B27 medium	Dorsomorphin, SB341542, XAV939, IGF-1, N2, B27, insulin, bFGF, DAPT, BDNF	PAX6, LHX2, MATH5, BRN3B, ISL1, TUJ1(TUBB3), NF-L, THY1, SNCG	40 days	Electro-physiological responses (patch-clamp recording)	45% (BRN3B+ ISL1+)	IHC
Langer et al. [52]	2018	hiPSC	EB, retinal organoid, RGC	NIM, RDM, BrainPhys Neuronal Medium	N2, B27, heparin, FBS	RGC: BRN3, ISL1, RBPMS, SNCG α-RGC: KCNG4, CB2, SPP1 DS-RGC: CART, CDH6, FSTL4	80 days	NA	NA	NA
Chen et al. [53]	2019	hiPSC	Neural spheres, RGC	RDM, RMM	IWR-1e, CHIR99021, SAG, N2, FBS, BDNF, RA, Y-27632	RPC: VSX2(CHX10) RGC: MATH5(ATOH7), BRN3B, TAU, NFM	34 days	NA	NA	NA
Chavali et al. [54]	2020	hiPSC	RPC, RGC	RPC induction medium, RGC induction medium	B27, N2, nicotinamide, XAV939, SB431542, LDN193189, IGF-1, bFGF, SHH, SAG, FGF8, follistatin, cyclopamine, DAPT, Y-27632, forskolin, cAMP, BDNF, NT4, CNTF	RPC: SOX2, RAX, PAX6 RGC: THY1/CD90, TUJ1, MAP2, BRN3A, BRN3B, RBPMS	36 days	Electro-physiological responses (patch-clamp recording)	58% (THY1+) 84% (BRN3B+) 12% (RBPMS+) After MACS: 95% (BRN3A+)	Flow cytometry After MACS: ICC
Rabesandratana et al. [55]	2020	hiPSC	Retinal organoid, RGC	Essential 6 medium, RDM	N2, B27, FGF2	THY1, BRN3A, PAX6, HuC/D, βIII-tubulin, RBPMS	63 days	Electro-physiological responses (patch-clamp recording, multi electrode array)	78.03 ± 1.47% (THY1+)	Flow cytometry
Gudiseva et al. [56]	2021	hiPSC	RPC, RGC	RPC induction medium, RGC induction medium	B27, N2, nicotinamide, XAV939, SB431542, LDN193189, IGF-1, bFGF, SHH, SAG, FGF8, follistatin, cyclopamine, DAPT, Y-27632, forskolin, cAMP, BDNF, NT4, CNTF	MAP2, RBPMS, TUJ1, BRN3A, SOX4, TUBB3 (βIII-tubulin), SNCG, PAX6, NRN1, CD90 (THY1)	40 days	NA	87% (BRN3+) 87% (SNCG+) 81% (THY1+) 19% (RBPMS+)	Flow cytometry
Vrathasha et al. [57]	2022	hiPSC	RPC, RGC	RPC induction medium, RGC induction medium	B27, N2, nicotinamide, XAV939, SB431542, LDN193189, IGF-1, bFGF, SHH, SAG, FGF8, follistatin, cyclopamine, DAPT, Y-27632, forskolin, cAMP, BDNF, NT4, CNTF	RPC: Ki67, VSX2(CHX10) RGC: BRN3/POU4F, SNCG, THY1/CD90, RBPMS	36 days	Electro-physiological responses (patch-clamp recording)	87% (BRN3+) 93% (SNCG+) 85.5% (THY1+) 22.5% (RBPMS+)	Flow cytometry
Luo et al. [58]	2022	hESC, hiPSC	RPC, RGC	RGC culture medium	B27, N2, FGF, insulin, sodium pyruvate, SATO supplement, L-glutamine, triiodothyronine, N-acetyl cysteine, BDNF, CNTF, forskolin, DAPT, GDNF	BRN3A, BRN3B, ISL1, SNCG, GAP43, ELAVL4 (HuD), ATOH7, RPBMS	1 week	Electro-physiological responses (calcium imaging)	NA	NA

ATOH7: atonal bHLH transcription factor 7; BDNF: brain-derived neurotrophic factor; bFGF: basic fibroblast growth factor; BRN3A: brain-specific homeobox/POU domain protein 3A; BRN3B: brain-specific homeobox/POU domain protein 3B; cAMP: cyclic adenosine monophosphate; CART: cocaine- and amphetamine-regulated transcript; CB2: cannabinoid receptor 2; CDH6: cadherin 6; CNTF: ciliary neurotrophic factor; DKK1: Dickkopf-related protein 1; DS-RGC: directionally selective retinal ganglion cell; EB: embryoid bodies; FBS: fetal bovine serum; FGF8: fibroblast growth factor 8; FSTL4: follistatin-related protein 1; GAP43: growth associated protein 43; GDNF: glial cell-derived neurotrophic factor; hESC: human embryonic stem cell; hiPSC: human-induced pluripotent stem cell; HuC/D: ELAVL protein homolog 4; ICC: immunocytochemistry; IGF-1: insulin-like growth factor 1; IHC: immunohistochemistry; ISL1: Islet1; KCNG4: potassium voltage-gated channel subfamily G member 4; LHX2: LIM homeobox protein 2; MACS: magnetic activated cell sorting; MAP2: microtubule-associated protein 2; mESC: mouse embryonic stem cells; NA: not available; NF-L: neurofilament light polypeptide; NFM: neurofilament medium polypeptide; NIM: neural- induction medium; NRN1: neuritin 1; NT4: neurotrophin-4; PAX6: paired box protein Pax-6; PIM: proneural induction medium; RAX: retina and anterior neural fold homeobox; RBPMS: RNA-binding protein with multiple splicing; RDM: retinal differential medium; RGC: retinal ganglion cell; RMM: retinal maturation medium; RPC: retinal progenitor cell; SAG: smoothened agonist; SHH: Sonic hedgehog; SNCG: gamma-synuclein; SOX2: SRY-box transcription factor 2; SPP1: secreted phosphoprotein 1; TAU: microtubule-associated protein tau; THY1: Thy-1 cell surface antigen; TUBB3: tubulin beta 3.

**Table 3:** Experimental features of photoreceptor differentiation.

Authors	Year	Cell Source	Differentiation Procedures	Differentiation Culture Media	Differentiation Factors	Immunostaining Markers	Time Length	Other Assays	Efficiency	Tests for Efficiency
Meyer et al. [19]	2011	hiPSC	Neural cluster, OV, photoreceptors	EB medium, NIM, RDM	Noggin, DKK1, N2, B27	OTX2, RCVRN, CRX, NRL	120 days	RT-qPCR, electro-physiological responses, calcium imaging	55.9 ± 6.6% (CRX+)	ICC
Zhong et al. [12]	2014	hiPSC	EF, NR, RC, photoreceptors	NIM	Heparin, N2, B27, taurine, RA	Precursor: OTX2, RCVRN Rod: RHO Cone: OPN1SW, OPN1L/MW	21 weeks	light responsiveness (electrical recording)	90% (RHO+)	IHC
Barnea-Cramer et al. [95]	2016	hiPSC	EFP, RNP, eyecup, PhRP, photoreceptors	RIM, NDM	Noggin, N2, B27, insulin, BDNF, CNTF, RA, DAPT	Progenitor: CRX, NRL, NR2E3 Rod: RHO, RCVRN, PDE6α	100 days	NA	NA	NA
Zhu et al. [96]	2018	hiPSC	NR, RO, photoreceptors	MEM, NSC medium	Noggin, DKK1, N1, IGF-1, IWR-1, SB431542, LDN193189	Precursor: OTX2, CRX, BLIMP1, AIPL1, RCVRN Rod: NRL Cone: THRB, ARR3, RXRγ	70 days	RT-qPCR	12 weeks: 52 ± 6% (OTX2+) 43 ± 3% (RCVRN+)	ICC
Ovando-Roche et al. [97]	2018	hiPSC	NRV, photoreceptor	NIM (in bioreactor)	N2, B27, taurine, RA	Precursor: RCVRN Rod: RHO, CD133, CD73 Cone: OPN1SW, OPN1L/MW, ARR3	17 weeks	NA	68.17 ± 6.15% (RCVRN+) 11.87 ± 1.88% (RCVRN+ CD73+)	flow cytometry
Gagliardi et al. [98]	2018	hiPSC	NR, RO	Essential 6 medium, ProB27 medium	N2 supplement, FGF2	Precursor: CRX, RCVRN, CD73 Rod: RHO Cone: OPN1SW, OPN1L/MW, ARR3	200 days	RT-qPCR, activity of CNG channels (calcium imaging)	55 ± 2% (CD73+)	flow cytometry
Li et al. [32]	2018	hiPSC	EB, NR, RO, photoreceptors	NIM, RDM	Heparin, N2, B27, taurine	Precursor: OTX2 Rod: RHO Cone: OPN1SW, OPN1L/MW	9 weeks	NA	NA	NA
Ribeiro et al. [99]	2021	hiPSC	RO, photoreceptors	Essential 6 medium, PIM, RDM	N2, B27, RA	Precursor: CRX, RCVRN Rod: RHO Cone: OPN1SW, OPN1L/MW	90 days	Electro-physiological responses (multi-electrode array), quantitation of light responsiveness	59 ± 3% (arrestin + OPN1L/MW+)	flow cytometry
Sanjurjo-Soriano et al. [37]	2022	hiPSC	NR, RO, photoreceptors	Essential 6 medium, RDM	N2, B27, FGF2, taurine, RA	Precursor: CRX, RCVRN Rod: RHO, NRL, NR2E3 Cone: OPN1SW, OPN1L/MW, ARR3	225 days	Western blot (PDE6B expression)	23.8–50.3% (ARR3+) 49.7–76.2% (RHO+)	IHC

AIPL1: aryl hydrocarbon receptor-interacting protein-like 1; ARR3: arrestin 3; BDNF: brain-derived neurotrophic factor; BLIMP1: B lymphocyte-induced maturation protein 1; CNG channel: cyclic nucleotide-gated ion channel; CNTF: ciliary neurotrophic factor; CRX: cone-rod homeobox; DKK1: Dickkopf-related protein 1; EB: embryoid bodies; EF: eye field; EFP: eye field progenitor; FGF2: fibroblast growth factor 2; hiPSC: human-induced pluripotent stem cell; ICC: immunocytochemistry; IGF-1: insulin-like growth factor 1; IHC: immunohistochemistry; MEM: minimum essential medium; NA: not available; NDM: neural differentiation medium; NIM: neural induction medium; NR: neural retina; NR2E3: nuclear receptor subfamily 2 group E member 3; NRL: neural retina leucine zipper; NRV: neuroretinal vesicle; NSC: neural stem cell; OPN1L/MW: Opsin 1 cone photoreceptors, medium- and long wave-sensitive; OPN1SW: opsin 1 cone photoreceptors, short-wave-sensitive; OTX2: orthodenticle homeobox 2; OV: optic vesicles; PDE6α: phosphodiesterase 6 alpha; PhRP: photoreceptor progenitor; PIM: proneural induction medium; RC: retinal cup; RCVRN: recoverin; RDM: retinal differential medium; RHO: rhodopsin; RIM: retinal induction medium; RNP: retinal neural progenitor; RO: retinal organoid; RT-qPCR: reverse transcription-quantitative polymerase chain reaction; RXRγ: retinoid X receptor gamma; THRB: thyroid hormone receptor beta.

## Data Availability

Not applicable.

## Abbreviations

3D: three-dimensional; AC: amacrine cell; AIPL1: aryl hydrocarbon receptor-interacting protein-like 1; AMD: age-related macular degeneration; AP2: activating protein 2; ARL13B: ADP-ribosylation factor-like protein 13B; ARR3: arrestin 3; ATOH7: atonal bHLH transcription factor 7; ATRA: all-trans retinoic acid; BC: bipolar cell; BDNF: brain-derived neurotrophic factor; bFGF: basic fibroblast growth factor; bHLH: basic-helix-loop-helix; BLIMP1: B lymphocyte-induced maturation protein 1; BMP4: bone morphogenetic protein 4; BRN3A: brain-specific homeobox/POU domain protein 3A; BRN3B: brain-specific homeobox/POU domain protein 3B; CALB: calbindin; cAMP: cyclic adenosine monophosphate; CAR: cone arrestin; CART: cocaine- and amphetamine-regulated transcript; CB2: cannabinoid receptor 2; CDH6: cadherin 6; CNG: cyclic nucleotide-gated; CNTF: ciliary neurotrophic factor; CRALBP: cellular retinaldehyde-binding protein; CRX: cone-rod homeobox; CTBP2: C-terminal-binding protein 2; DKK1: Dickkopf-related protein 1; DS-RGC: directionally selective retinal ganglion cell; EB: embryoid body; EF: eye field; EFP: eye field progenitor; ESCs: embryonic stem cells; FBS: fetal bovine serum; FGF2: fibroblast growth factor 2; FGF8: fibroblast growth factor 8; FSTL4: follistatin-related protein 1; GABA: gamma-aminobutyric acid; GAD2: glutamic acid decarboxylase 2; GAP43: growth-associated protein 43; GDNF: glial cell-derived neurotrophic factor; GLAST: glutamate aspartate transporter; GNAO1: G protein subunit alpha O1; GNAT1: G protein subunit alpha transducin 1; GRK1: G protein-coupled receptor kinase 1; HC: horizontal cell; hESCs: human embryonic stem cells; hiPSCs: human induced pluripotent stem cells; HuC/D: ELAVL protein homolog 4; ICC: immunocytochemistry; IGF-1: insulin-like growth factor 1; IHC: immunohistochemistry; ipRGCs: intrinsically photosensitive RGCs; iPSCs: induced pluripotent stem cells; ISL1: Islet1; IWR-1: inhibitor of Wnt response 1; KCNG4: potassium voltage-gated channel subfamily G member 4; LHX2: LIM homeobox protein 2; LIM1: LIM homeobox protein 1; MACS: magnetic-activated cell sorting; MAP2: microtubule-associated protein 2; MC: Muller cell; MCM2: minichromosome maintenance complex component 2; MEM: minimum essential medium; MITF: microphthalmia-associated transcription factor; NA: not available; NDM: neural differentiation medium; NEUROD1: neurogenic differentiation factor 1; NF-L: neurofilament light polypeptide; NFM: neurofilament medium polypeptide; NGN2: neurogenin 2; NIM: neural-induction medium; NR: neural retina; NR2E3: nuclear receptor subfamily 2 group E member 3; NRL: neural retina leucine zipper; NRN1: neuritin 1; NRV: neuroretinal vesicle; NSC: neural stem cell; NT4: neurotrophin-4; OC1: one cut domain, family member 1; OCT: optical coherence tomography; OPN1L/MW: opsin 1 cone photoreceptors, medium- and long wave-sensitive; OPN1SW: opsin 1 cone photoreceptors, short wave-sensitive; OPN4: melanopsin; OTX2: orthodenticle homeobox 2; OV: optic vesicles; PAX6: paired box protein Pax-6; PBG: polybenzyl glutamate; PCNT: pericentrin; PDE6α: phosphodiesterase 6 alpha; PhRP: photoreceptor progenitor; PIM: proneural induction medium; PKCα: protein kinase C alpha; PROX1: prospero homeobox protein 1; PSCs: pluripotent stem cells; qPCR: quantitative real-time polymerase chain reaction; RA: retinoic acid; RAX: retina and anterior neural fold homeobox; RBPMS: RNA-binding protein with multiple splicing; RC: retinal cup; RCVRN: recoverin; RDM: retinal differential medium; RGCs: retinal ganglion cells; RHO: rhodopsin; RIM: retinal induction medium; RLBP1: retinaldehyde-binding protein 1; RMM: retinal maturation medium; RNP: retinal neural progenitor; RO: retinal organoid; RP: retinitis pigmentosa; RPCs: retinal progenitor cells; RPE: retinal pigment epithelium; RT-qPCR: reverse transcription-quantitative polymerase chain reaction; RXRγ: retinoid X receptor gamma; SAG: smoothened agonist; scRNA-seq: single-cell RNA sequencing; Shh: Sonic hedgehog; SIX3: sine oculis homeobox homolog 3; SIX6: sine oculis homeobox homolog 6; SNCG: gamma-synuclein; SOX1: SRY-box transcription factor 1; SOX2: SRY-box transcription factor 2; SOX9: SRY-box transcription factor 9; SPP1: secreted phosphoprotein 1; TAU: microtubule-associated protein tau; TBR2: T-box brain protein 2; TEM: transmission electron microscopy; TGF-β: transforming growth factor beta; THRB: thyroid hormone receptor beta; THY1: Thy-1 cell surface antigen; TUBB3: tubulin beta 3; VGLUT1: vesicular glutamate transporter 1; VSX2: visual system homeobox 2.
